# Help Is Just a Message Away: Online Counselling Chat Services Bridging Gaps in Youth Mental Health?

**DOI:** 10.3390/ejihpe15120257

**Published:** 2025-12-15

**Authors:** Alexis Dewaele, Elke Denayer, Maria Cabello, Irati Higuera-Lozano, Tuuli Pitkänen, Katalin Felvinczi, Zsuzsa Kaló, Siiri Soininvaara, Lien Goossens

**Affiliations:** 1 Department of Experimental Clinical and Health Psychology, Faculty of Psychology and Educational Sciences, Ghent University, 9000 Gent, Belgium; 2 Department of Psychiatry, Universidad Autónoma de Madrid, 28029 Madrid, Spain; 3 Instituto de Salud Carlos III, Centro de Investigación Biomédica en Red de Salud Mental (CIBERSAM), 28029 Madrid, Spain; 4 Finnish Youth Research Society, 00520 Helsinki, Finland; 5 Institute of Psychology, ELTE Eötvös Lóránd University, 1064 Budapest, Hungary; 6 Department of Developmental, Personality and Social Psychology, Faculty of Psychology and Educational Sciences, Ghent University, 9000 Gent, Belgium

**Keywords:** digital support, online counselling chat services (OCCS), service evaluation, stakeholder perspectives, youth mental health

## Abstract

Adolescents and young adults across Europe face growing mental health challenges, yet many do not seek professional help. Online counselling chat services (OCCS) offer anonymous, accessible, and youth-friendly support, but their varied aims, formats, and resources complicate evaluation and integration into formal care systems. This study aimed to identify shared priorities for the development, evaluation, and implementation of OCCS for youth. Eight focus groups were conducted with 38 stakeholders—including researchers, counsellors, and service coordinators—from eight European countries. Through qualitative content analysis, six key thematic domains emerged: usability and engagement, service quality and effectiveness, infrastructure and integration, sustainability, ethical considerations, and future visions. Participants highlighted OCCS as valuable tools for fostering emotional safety, trust, and accessibility, while also noting persistent challenges such as limited funding, fragile infrastructure, and ethical tensions around anonymity and safeguarding. Crucially, the need for flexible evaluation frameworks that reflect service diversity and for stronger cross-model collaboration was emphasized. These findings provide a strategic foundation for advancing inclusive, sustainable, and youth-centered digital mental health support across Europe.

## 1. Introduction

Mental health challenges among adolescents and young adults represent a pressing public health concern. Approximately 75% of mental health difficulties emerge before the age of 24, and 50% before the age of 14 (James et al., 2018). These issues make up nearly 45% of all the years of healthy life lost among young people under 25 (Gore et al., 2011; Murray, 2022). Despite the high prevalence and long-term consequences of untreated mental health conditions—including academic underachievement, reduced life satisfaction, and increased risk of chronic disorders—only a small proportion of adolescents and young adults access professional help (Essau, 2005; Radez et al., 2021). While these challenges are global, their manifestation and impact vary significantly across regions, social groups, and individual circumstances. In Europe, recent crises such as the COVID-19 pandemic, the war in Ukraine, and climate change have intensified uncertainty and negatively affected youth mental health (Badanta et al., 2024; Hickman et al., 2021).

Online counselling chat services (OCCS) might help to fill gaps in mental health provision—offering real-time, text-based support that aligns with how many young, digitally native people prefer to communicate (Higuera-Lozano et al., 2025). However, ‘OCCS’ is a broad term: large, professional services often aim to complement formal care, whereas smaller or voluntary services may primarily provide non-clinical, on-demand conversations. This diversity has important implications for how OCCS are defined, evaluated, and positioned within broader systems of support. To date, there is little agreement on consistent frameworks for evaluation or integration. This study focuses on OCCS within a European context, drawing on data from Belgium, Finland, Hungary, and Spain. These countries represent diverse cultural and structural approaches to youth mental health and digital support, allowing for insight into shared challenges and best practices. Within this specific context, we focus on synthesizing perspectives on the roles, limitations, and future directions of OCCS—particularly those with professional capacity—to inform development and policy. By analyzing perspectives across distinct service models and cultural contexts, this study contributes to a cross-national understanding of OCCS practices that has been largely absent in prior literature and offers concrete directions for strengthening youth-oriented digital mental health care.

### 1.1. Supporting Youth Mental Health via Online Counselling Chat Services

The vulnerability of adolescents and young adults to mental health issues is rooted in a complex interplay of biological, cognitive, and psychosocial changes. Psychosocially, youth navigate critical developmental tasks such as identity formation and the establishment of social relationships (Brown & Larson, 2009; Meeus et al., 2010). These developmental dynamics are compounded by systemic barriers to care. Radez et al. (2021) identify four categories of barriers: individual (e.g., lack of knowledge), social (e.g., stigma), relational (e.g., confidentiality concerns), and systemic (e.g., long waiting times). Digital interventions offer a way to circumvent many of these barriers by providing accessible, anonymous, and flexible support.

OCCS are a form of human-supported digital intervention delivered via synchronous text-based communication. These services respond to diverse youth needs—ranging from seeking information and a safe space to talk about personal or developmental issues (Takala et al., 2025) to coping with acute crises, including suicidal thoughts, which remain a frequent topic of conversations (Helfer et al., 2025). These services can enhance young people’s agency by providing conversational support, service counseling, and assistance alongside or between face-to-face care, while also facing challenges in recognizing severe cases and ensuring appropriate referral and collaboration with professional services.

They are typically staffed by trained volunteers or mental health professionals and are designed to provide immediate, anonymous support (Barak et al., 2009; Tibbs et al., 2022). These services are particularly appealing to youth because they align with familiar communication styles, are silent and discreet, can be accessed from any location, and are often available outside office or school hours (Garrett et al., 2023; Vanhove & Vercaigne, 2011). The benefits of OCCS extend beyond users. For counsellors, the written format allows for reflection and boundary-setting, while organizations benefit from lower operational costs and the ability to reach larger populations. OCCS also generates valuable data for service improvement and research (Child Helpline International, 2023).

However, OCCS are not without limitations. Technical issues, lack of non-verbal cues, and the potential for miscommunication can hinder the supportive process (Baker & Ray, 2011). Additionally, structural challenges such as limited funding, staffing shortages, and visibility issues persist (Mathieu et al., 2021; Sindahl et al., 2020). A recent study (Higuera-Lozano et al., 2025) also identified key challenges for strengthening OCCS across Europe, including limited service hours, data security concerns, gaps in support for vulnerable groups, insufficient multilingual access, and the need for improved data reporting and provider collaboration. Finally, there is considerable diversity among OCCS: some are large professional services, while others are smaller NGO-run or volunteer-based initiatives, differing in aims, opening hours, capacity, and target groups. The recent mapping of 71 services across four European countries showed that most were provided by NGOs, many combined chats with helplines, only a small minority operated 24/7 or offered support in minority languages, and approaches ranged from topic-specific expertise to general listening support (Higuera-Lozano et al., 2025). This diversity has important implications, as existing evaluation frameworks may need to be adapted to account for the different aims, capacities, and roles of these services rather than applying uniform criteria across all OCCS.

### 1.2. Evaluating Online Counselling Chat Services: Key Insights from Existing Frameworks

A growing body of literature has developed frameworks to guide the evaluation and implementation of digital mental health interventions. These frameworks consistently emphasize six key domains:

(1) Privacy and security: Ensuring data protection and transparency (e.g., Zelmer et al., 2018).
(2) Clinical validity: Emphasis on evidence-based practice and therapeutic effectiveness (e.g., Agarwal et al., 2022).
(3) Usability and engagement: Prioritizing user-centered design and accessibility (e.g., Martin et al., 2014; Lagan et al., 2021).
(4) Technical aspects: Addressing interoperability and functionality (e.g., Torous et al., 2021).
(5) Economic considerations: Evaluating cost-effectiveness and resource requirements (e.g., Villarreal-Zegarra et al., 2021).
(6) Ethical considerations: Promoting equity and cultural appropriateness (e.g., Ramos et al., 2021).

These frameworks also highlight critical implementation factors. Resource allocation is a recurring theme, with studies noting that insufficient investment can hinder scalability (Berardi et al., 2024). Training and support for providers are essential, as are meaningful stakeholder engagement and referral pathways, including youth voices and stigmatized communities (Zelmer et al., 2018; Ramos et al., 2021).

Accessibility and equity are also central concerns. While OCCS can reduce barriers for many, they may inadvertently exclude others—such as youth without private internet access or those with limited literacy (Mathieu et al., 2021). Frameworks increasingly call for inclusive design and evaluation practices that account for diversity in user needs and contexts.

There is also a growing push for standardization. Lipschitz et al. (2022) propose harmonized reporting of engagement metrics, while Lagan et al. (2021) advocate for integrated frameworks that balance usability with clinical impact. This shift reflects a broader trend toward evidence-based, system-level thinking, recognizing that successful implementation depends on alignment across individual, organizational, and policy levels (Torous et al., 2021; Berardi et al., 2024).

### 1.3. Towards a Consensus-Based Understanding

The current landscape of OCCS for youth is highly fragmented. Services vary widely in scope, resources, and aims, yet there is little agreement on how they should be defined, evaluated, or integrated into broader mental health systems. Existing evaluation frameworks for digital mental health interventions provide useful principles, but they rarely address the specific features of synchronous, text-based counselling or the unique developmental needs of adolescents and young adults. This lack of shared understanding creates both a practical and conceptual gap: without clarity on the roles and standards of OCCS, it is difficult to ensure quality, equity, and sustainability across services.

The study’s aim is to contribute to building consensus by identifying shared priorities and areas of dissensus among experts from research and applied practice. OCCS for adolescents and young adults are understood here as a diverse set of text-based services, ranging from professional counselling to volunteer-run, low-threshold support. Our goal is not to assess the effectiveness of these services in general but to identify key thematic areas and priorities that should guide their future development. To this end, we draw on insights from in-person and online focus groups held within the framework of the European CHAT-YOUTH project. The guiding research question is therefore: What are the priority areas for research, practice, and policy to support the future development of OCCS for youth in Europe, as identified by experts from research and applied practice? By addressing this question, the study seeks to provide a strategic roadmap that highlights where consensus can be built, where important dissensus remains, and how these insights can inform the design, evaluation, and implementation of high-quality OCCS for young people. Taken together, these elements highlight a critical gap in the field: although OCCS are rapidly expanding as first-line youth support, systematic knowledge of how different stakeholders envision their functioning, challenges, and development priorities remains limited. Our study addresses this gap by synthesizing diverse practitioner perspectives into a coherent, empirically grounded set of priorities for OCCS development, evaluation, and implementation. 

## 2. Materials and Methods

The CH@T-YOUTH project (Crisis Help and Assistance for Youth during Challenging Times) is a cross-country collaborative project that covers four European countries (Finland, Spain, Hungary, and Belgium). The project aims to identify the characteristics, strengths, and best practices of OCCS that support young people in crisis. Our study builds on two earlier investigations conducted within the CHAT-YOUTH project. The first study mapped 71 online counselling chat services (OCCS) across four European countries, highlighting substantial diversity and several gaps in meeting established quality standards (Higuera-Lozano et al., 2025). The second study gathered perspectives from counsellors and coordinators of 33 OCCS, identifying key benefits, limitations, and potential solutions for improving service delivery (Higuera-Lozano et al., in review). Building on these findings, the present study seeks to address the identified shortcomings by engaging a broader range of stakeholders to develop consensus-based insights into the future roles, priorities, and implementation of OCCS for young people.

### 2.1. Recruitment and Procedure

A total of four in-person focus groups (May 2025) and four online focus groups (June 2025) were conducted, each lasting approximately 90 min. The focus groups were facilitated by a team of five researchers (A.D., L.G., T.P., and K.F. for the in-person groups; A.D., L.G., T.P., and E.D. for the online groups), whose combined expertise spans clinical, developmental, and youth psychology, as well as online counselling and mental health. The team brought substantial academic and practical experience to the discussions, ensuring both methodological rigor and sensitivity to the subject matter. Groups consisted of 3–8 participants to balance diversity and manageability. The in-person focus groups (*n* = 27) followed a semi-structured format based on a topic list (see Supplementary Annex S1) that included six overarching themes: (1) usability, accessibility & user engagement, (2) effectiveness and service quality, (3) infrastructure and integration within broader care systems, (4) sustainability and resource models, (5) ethical considerations and equity, and (6) future visions for OCSS (cf. the six key domains identified in the introduction of this paper, here slightly adapted). Each theme included specific questions exploring challenges, opportunities, and best practices. Between the two rounds of focus groups, consensus statements were derived by the main researchers based on the transcripts of the in-person sessions. These statements were then shared with the participants of the online focus groups (*n* = 23) in advance via e-mail and further discussed during the online meetings.

Participants for the focus groups were recruited based on the mapping exercise from the first study and in collaboration with Child Helpline International. The latter disseminated invitations to its European member network. Potential participants first completed a short registration form indicating availability and interest. Those confirming attendance received an information letter and informed consent form. The study was approved by the Ghent University Ethical Committee of the Faculty of Psychology and Educational Sciences. Informed consent included explicit agreement to audio recording and transcription, with assurances of voluntary participation and the right to withdraw without consequence. For individuals declining data use consent, alternative non-recorded participation was offered. Participants provided demographic information, including age, nationality, and professional background. All transcripts were audio-recorded, transcribed verbatim, and pseudonymized. The latter involved removing or altering information that could directly or indirectly lead to identification of individuals.

The inclusion criteria required participants to be actively engaged in, or conducting research on, online chat counseling services. Eligible individuals included volunteer or professional counselors, managers, staff members, and researchers.

The sample (*n* = 38, see Table 1) comprised participants from six European countries (Belgium, Spain, the Netherlands, Finland, Poland, and Hungary), as well as from Gibraltar and the United Kingdom. Participants included researchers (*n* = 15), OCCS chat counsellors (*n* = 7), and OCCS management or staff members (*n* = 9). Other participant profiles (*n* = 7) included a project officer in youth mental health, an NGO executive director and project coordinator, a trainer in online and blended support, a master’s student in psychology, a student mentor, and a policymaker. Given the targeted sampling approach, no specific exclusion criteria were applied. As this study employed qualitative deductive content analysis, no formal sample size calculations were conducted. Instead, the sample size was determined based on the principle of information power (Malterud et al., 2016), ensuring sufficient variation in stakeholder roles and national contexts to support meaningful thematic exploration.

The composition of the sample reflects both the recruitment strategy and the availability of participants across Child Helpline International’s European network. As a result, representation was uneven across countries and professional roles, with higher participation from Belgium and lower participation from several other member countries. This imbalance stemmed from differences in service size, language accessibility (all group discussions were in English), and responsiveness during recruitment. Given that the study aimed to capture a broad range of perspectives rather than achieve statistical representativeness, this distribution was considered appropriate for our purposes, especially given the fact that earlier work within the project already gathered information in a broader sample (see Higuera-Lozano et al., 2025). The overrepresentation of certain contexts provided rich, in-depth insight into more established service models, while contributions from smaller or less-resourced services and countries still enabled comparative reflection.

### 2.2. Data Analysis

Data from both in-person and online focus groups were analyzed using directed (deductive) qualitative content analysis (see e.g., Kibiswa, 2019), guided by a predefined categorization matrix. This approach allowed us to organize stakeholder input within a structured framework while still enabling inductive refinement where the data warranted it, supporting both consistency and sensitivity to contextual nuance. This matrix contains 29 codes with clear operational definitions (see Table 2) to reduce coder variability.

#### Coding and Validation Strategy

The coding process was conducted by a single researcher (Dewaele) using Microsoft CoPilot (bizchat.20251204.45.7 version) to support the identification of relevant text segments across eight focus group transcripts. Through a university-wide agreement with Microsoft, all data entered via CoPilot are protected under enterprise data security measures. CoPilot was configured to detect segments potentially corresponding to the predefined coding scheme. Preliminary codings were systematically entered, refined, and organized with the software package MAXQDA 24 (Analytics pro) to support structured analysis and ensure transparency in the coding process. Importantly, all transcripts were read and reviewed manually by the researcher both before and after the automated identification process to ensure alignment with the operational definitions of the codes. This included removing codes from 174 segments that were overly inclusive; for example, those only tangentially related to the intended theme, those that fit better under another code, and codes applied to moderator interventions or to introductory and closing remarks. For example, one quote described youth attempting to access chat services but disengaging due to long waiting times. CoPilot initially assigned this segment to the code “Youth participation” (i.e., involving youth in co-design, feedback, or outreach), which was deemed inaccurate or overly inclusive. Consequently, the segment was excluded from the final coding. The researcher re-read the transcripts in full and added codes to 13 additional segments that had been missed, ensuring thorough manual control and contextual accuracy. In total, the 29 codes were applied 995 times across all eight transcripts (see Table 3).

As coding was conducted by a single researcher, inter-rater reliability was not applicable in this context. Instead, validity was strengthened through multiple strategies: drawing on prior CHAT-YOUTH studies (Higuera-Lozano et al., 2025) and using Microsoft CoPilot as an independent tool for systematic segment identification. To further enhance the reliability of the coding, the researcher engaged in a reflexive validation process, which included the use of reflective and analytic memos (Finfgeld-Connett, 2014) to document coding decisions, uncertainties, and rationale. The coding scheme was iteratively refined to ensure consistent application across all transcripts. Finally, the results were cross-validated through a member check (see Erdmann & Potthoff, 2023): five focus group participants (one researcher, two OCCS staff members, and two representatives from an international NGO for child and youth helplines) provided feedback and were informed about whether and how their feedback was addressed. To further assess the internal consistency of the coding, a co-occurrence matrix was generated using MAXQDA’s Code Relations Browser. The matrix (see Supplementary Annex S4) revealed substantial overlap between conceptually related codes—such as “Bonding & Alliance” and “Trust & Credibility,” and “Training & Supervision” and “Volunteer Engagement”—supporting the thematic coherence of the analysis. These relationships reflect expected intersections within the predefined coding framework and reinforce the validity of the deductive content analysis approach. As such, the combination of CoPilot-assisted identification, manual validation, reflexive documentation, member checking, and investigating code relations provides a robust foundation for the credibility and trustworthiness of the findings. Each of the six thematic areas was structured around subtopics (conforming to the predefined categorization matrix that includes 29 subtopics in total) and validated through recurrence and emphasis in the data. The analysis integrates quotes that illustrate key tensions and opportunities, grounding the results in authentic practitioner voices and lived experience across varied European contexts. In the results section, hashtags indicate the number of codes assigned to a specific subtopic. Quotes were slightly adjusted for readability and preserving anonymity. To best ensure anonymity, only the professional role of the quoted participant is reported.

## 3. Results

This section presents findings from a thematic synthesis of coded segments across eight focus groups.

### 3.1. Usability, Accessibility & User Engagement

Participants discussed five interrelated dimensions that shape how youth in Europe engage with OCCS: inclusive design (#43), youth participation (#25), stigma and misconceptions (#23), user experience (#43), and communication preferences (#33).

Participants praised OCCS for their potential to reach, amongst others, marginalized, neurodiverse, and low-literacy youth. Features such as speech-to-text, AI translation, and simplified interfaces were seen as having potential to make services more accessible. Chat services were described as low-barrier, anonymous, and adaptable to different cultural and linguistic contexts. However, concerns were raised about over-labeling user groups, with some advocating for universal design principles that benefit all users. One participant noted, “We prioritized easy access in our chat service, especially for youth with autism or suicidal thoughts, who tend to leave if the entry process is too demanding. So we minimized pre-chat questions—just age confirmation—and allowed users to chat without participating in research unless they chose to. This low-barrier design was essential to ensure inclusion and comfort.” (OCCS staff member).

While youth feedback was gathered by some OCCS through post-chat surveys, structured co-design was mentioned only once. Some organizations involved youth councils or volunteer groups in service development, while others used youth-led outreach or referred to the importance of youth peer-to-peer support models. Participants emphasized the need to consult youth directly, especially regarding AI integration and service branding. Despite enthusiasm, practical challenges—such as time and resource constraints—were acknowledged.

Participants noted that some users had misunderstandings about OCCS. Some youth assumed chat services were only for crises or serious problems, leading to disengagement. Cultural stigma around mental health, gendered perceptions of help-seeking, and lack of inclusive branding further limited access. Services targeting LGBTQ+ youth or using diverse imagery were seen as more effective in countering these misconceptions. One participant reflected, “We found many youth were regular callers, and some didn’t identify with the term ‘child,’ so we created a separate teen line. … We’ve learned that inclusive naming and branding—like offering both child and teen lines—can make services feel more relevant and accessible.” (OCCS manager).

Ease of use may be a major factor in engagement. Youth were perceived to value minimal onboarding, intuitive navigation, and flexible feedback systems (e.g., emoji ratings, open surveys). Long wait times and survey fatigue were common barriers. Services that offered example prompts, asynchronous access, and low-pressure entry points were seen as more effective. Universal design and mobile accessibility were emphasized as key to satisfaction.

Youth were perceived to prefer chat over phone or in-person formats due to its anonymity, discretion, and emotional safety. Some participants mentioned that preferences could vary by gender and neurodiversity, with non-binary youth and users with autism favoring written communication. Informal and reflective interactions may be particularly engaging for youth. Participants stressed the importance of user autonomy—allowing youth to choose between human and bot interactions—and tailoring communication to their comfort and context.

### 3.2. Effectiveness and Service Quality

Participants across the eight focus groups discussed the five key dimensions shaping the effectiveness and quality of OCCS for youth in Europe: bonding & alliance (#41), engagement and continuity (#38), training & supervision (#56), measuring impact (#43), and ghosting & dropout (#20).

Bonding and alliance were consistently described as foundational to OCCS. Participants emphasized that youth often seek to feel heard and validated more than to receive solutions. Anonymity, non-judgmental listening, and emotional safety were seen as critical enablers of trust. Several services train volunteers specifically in empathy and motivational interviewing to foster alliance. One participant noted, “For us, the main goal in a crisis or first contact is bonding. We train our volunteers extensively in this. When the bond is strong, youth often share their phone number, which allows us to follow up—like, ‘Next week, we’ll talk again.’ That’s when we know it’s working. We’ve tried sending forms or links, but they rarely respond. If they trust us enough to give their number, that’s success. And we always tell them they’re in control—they can block us or end the process anytime. We’re here as long as they want to talk.” (chat-counsellor).

Maintaining user involvement over time was seen as both a strength and a challenge. Repeat visits were interpreted by some participants as signs of trust and service value. Some services use structured follow-up (e.g., scheduled calls), while others rely on open-ended surveys or warm referrals (i.e., a referral process in which the service provider actively facilitates the client’s connection to another service). Embedding OCCS in online community platforms or using peer-to-peer models was also seen as a way to sustain informal engagement. However, concerns about dependency and ethical boundaries were raised. It is important to note, however, that many OCCS are designed for one-off, anonymous consultations and do not have the mandate or capacity for follow-up; in such cases, fostering ongoing user involvement is neither expected nor intended, reflecting a different service philosophy than models that emphasize continuity.

High-quality training and supervision were viewed as essential to service effectiveness, not only for new counsellors but also through ongoing training and education for experienced staff. Programs varied from short onboarding to year-long curricula, sometimes including role-play, thematic modules, and real-time supervision. Emotional debriefing and peer support were emphasized to prevent burnout. Notably, several participants reported no difference in user feedback between trained volunteers and professionals, highlighting the importance of relational skills over formal education.

Participants widely acknowledged the difficulty of assessing impact due to anonymity and one-off interactions. While some services use pre-/post-chat ratings or satisfaction surveys, these were sometimes seen as biased or incomplete. AI tools and chat log analysis were emerging as promising methods for identifying patterns and outcomes. Still, many called for a redefinition of effectiveness—shifting from long-term outcomes to immediate emotional relief and perceived support or, as noted by one participant: “…maybe we need to redefine what effectiveness means for chat services. Instead of relying on traditional research models—like time-one and time-two measurements—we might need a new framework that fits the realities of anonymous, real-time support.” (researcher)

Dropout was a pervasive concern, especially in anonymous contexts where users may disengage mid-chat or never return. Long wait times, lack of trust, or emotional overwhelm were cited as contributing factors. Some services experimented with AI automated chatbots in waiting rooms, but these were only perceived as effective when initial bonding had occurred. Separately, participants noted the emotional toll on counsellors of not knowing what happened to a user after the conversation ended.

### 3.3. Infrastructure and Integration Within Broader Care Systems

Participants discussed five key dimensions shaping the infrastructural and systemic robustness of OCCS for youth in Europe: technological innovation (#34), platform stability (#14), interoperability (#26), cross-national implementation (#35), and integration into care pathways (#30).

Participants highlighted a range of non-AI tools that can enhance OCCS functionality. These included speech-to-text and text-to-speech features for low-literacy users, embedded chat modules on partner websites, and structured feedback systems (e.g., emoji ratings, session rating scales). Shared platforms and backend tools for anonymized data tracking were also seen as valuable. Innovations such as integration with widely used messaging platforms and warm handover protocols were developed in response to user needs. It helps us show progress, especially in crisis cases. We’re also exploring anonymous surveys via our website and social media to gather more feedback without disrupting the chat experience.” (OCCS manager).

Technical reliability was a recurring concern. Services reported challenges with server overload during crisis surges, connectivity issues, and accessibility barriers for users with disabilities. Shared infrastructure, while cost-effective, posed risks of cascading failures. Real-time availability indicators and backend databases were used to manage uptime and ensure continuity. Participants emphasized that technical glitches—such as slow response times—can significantly impact user trust and satisfaction.

Interoperability with local systems was perceived as beneficial for effective service delivery. Some chat services were integrated with clinical records, child protection databases, or emergency protocols, enabling smoother referrals and faster responses. Still, participants noted that feedback loops between referring and receiving organizations were often missing, limiting continuity of care. A second aspect concerned cooperation between chat service providers themselves. Shared software platforms, warm referrals, and real-time operator status tools facilitated coordination across services. One participant illustrated the advantages and drawbacks of such systems: “We use a shared chat software across 15 organizations in our region, which allows us to co-develop features like translation tools at a much lower cost. If one organization builds something useful, others can adopt it without paying separately. The downside is that if the server goes down, it affects everyone” (OCCS coordinator).

The implementation of and collaboration between OCCS across Europe presented significant challenges. Legal differences around anonymity, mandatory reporting, and data sharing created barriers to standardization. Language diversity and cultural norms further complicated service design and outreach. Participants called for shared platforms, multilingual support, and flexible protocols that respect local contexts. While pan-European collaboration was seen as aspirational, many emphasized the need for localized relevance and adaptability.

Finally, OCCS were increasingly viewed as entry points into broader mental health systems. Services facilitated referrals to therapists or more specialized OCCS, emergency responders, and community resources. Structured follow-up, safety planning, and case management systems supported continuity of care. However, anonymity and user autonomy sometimes limit deeper integration. Participants stressed the importance of balancing ethical safeguards with effective triage and referral mechanisms.

### 3.4. Sustainability and Resource Models

Participants discussed five interrelated dimensions that shape the long-term viability of OCCS for youth in Europe: funding models (#26), volunteer engagement (#47), staffing and capacity (#32), scalability (#29), and pan-European collaboration (#33).

Sustainable funding remains a central challenge. Many services rely heavily on government support, which can be unstable and temporary and tied to complex reporting requirements. Participants emphasized the need to demonstrate measurable impact to secure funding, often through outcome metrics or pilot projects. Hybrid models—combining public, private, and community-based funding—were by some seen as more resilient. One participant noted, “To build secure, fit-for-purpose systems, you need reliable funding—not just for tech infrastructure, but also to train staff. Free tools sometimes raise serious privacy concerns, especially when youth share sensitive issues. Without proper investment, it’s hard to ensure data protection and ethical service delivery.” (NGO project coordinator). Shared infrastructure and cost-efficient tools were also proposed to reduce financial strain.

Volunteers are the backbone of many OCCS, but recruitment and retention require structured support. Participants from services with comprehensive training, supervision, and emotional debriefing reported that this enables retention. Building a sense of community and offering flexible roles—such as outreach or design—were seen as effective strategies. Some services integrated student volunteers or older adults, while others faced challenges balancing specialization and generalization. Volunteers’ emotional investment was highlighted as both a strength and a vulnerability.

Staffing shortages and burnout were recurring concerns. High demand, especially during crises, often outpaced available personnel. Night shifts, emotional strain, and lack of supervision contributed to attrition. Flexible staffing models, time zone-based coverage, and counsellor support systems were proposed to mitigate these issues. Participants stressed the importance of maintaining institutional knowledge and investing in scalable training tools. One participant reflected, “After a school shooting, our chat service was overwhelmed—four times the usual volume. We urgently called on volunteers, and many responded, even without direct contact. Some said helping eased their own anxiety. The experience led us to develop a better protocol for future crises, including ways to contact and support volunteers, especially those returning after a long break.” (OCCS staff member).

Expanding OCCS to reach more youth required both technical and organizational innovation. Services explored embedding chat modules in popular online and social media platforms, leveraging AI for translation and triage, and partnering with academic institutions to increase volunteer capacity. However, cultural and linguistic barriers, limited infrastructure, and uneven funding were mentioned to pose challenges. Decentralized models and local adaptation were favored by some over centralized national systems.

Cross-national collaboration was seen as both necessary and complex. Shared platforms, standardized training, and a framework of good practices (including suitable evaluation tools) were proposed to improve efficiency and comparability. Yet, legal differences, cultural norms, and funding disparities limited integration. Participants called for flexible models that respect local contexts while enabling knowledge exchange and joint development. The idea of a unified European infrastructure was aspirational but tempered by concerns about feasibility and cost.

### 3.5. Ethical Considerations and Equity

Participants discussed four key dimensions that shape the ethical integrity and crisis responsiveness of OCCS for youth in Europe: anonymity vs. safety (#33), equity & inclusion (#36), safeguarding protocols (#36), and data privacy & consent (#34).

Balancing user privacy with emergency response emerged as a central ethical tension. While anonymity is foundational to OCCS, participants acknowledged that in life-threatening situations, confidentiality may need to be breached. National protocols varied widely—some services required user consent before contacting emergency services, while others acted unilaterally in high-risk cases. Technical tools like IP tracking were used cautiously, with legal constraints shaping their application. One participant explained, “In one-to-one sessions, I always explain that confidentiality is guaranteed—except if there’s a risk to life. That informed consent is crucial, and the same applies to chat services. We don’t contact emergency services just for suicidal thoughts; we triage and act only if there’s an immediate plan. If escalation is needed, we inform the user and have systems in place, like IP tracking, while respecting legal boundaries.” (NGO project coordinator). Emotional strain on staff due to lack of closure or inability to follow up was also noted. Techniques for good endings could be further explored through practitioner exchange.

Ensuring fair access for all youth was seen as both a moral imperative and a design challenge. Services highlighted the need for low-barrier entry, multilingual support, and culturally sensitive communication. Some groups—including LGBTQ+ youth, migrants, neurodiverse individuals, and those in rural areas—were perceived to face unique barriers in relation to the support they need. Inclusive training, diverse representation in counsellor profiles, and outreach via familiar online community platforms were proposed to improve engagement. One participant emphasized, “For many youth, just being heard is enough. We’ve seen countless transcripts where young people say, ‘I’ve never told this to anyone.’ That alone shows the value of chat services in reaching those who don’t access traditional support. It’s a question of accessibility, diversity, and creating space for underserved voices.” (researcher).

Safeguarding procedures varied across services but shared a common goal: protecting youth while maintaining trust. Structured protocols for suicide risk, violence, and abuse were mentioned, often involving shift supervisors or interagency collaboration and referral mechanisms. Training in psychological first aid and crisis response was emphasized, especially given the lack of visual or vocal cues in chat-based formats. Some services used warm handovers and feedback loops to ensure continuity of care. Ethical prioritization—such as “life over confidentiality”—was a guiding principle, though legal and cultural contexts shaped its implementation.

Participants stressed the importance of transparent data practices and informed consent. Most participants referred to chat services operating under strict anonymity, with minimal data collection and GDPR-compliant systems. Consent was required before sharing information or escalating cases. AI tools raised new concerns about data retention, transparency, and ethical use. Services emphasized the need to clearly inform users when interacting with bots and to ensure that data remains within secure jurisdictions.

### 3.6. Future Visions for OCSS

Participants discussed five interrelated dimensions shaping the future of OCCS for youth in Europe: Youth-centered Innovation (#29), trust and credibility (#41), long-term vision and ideal models (#29), adaptability (#34), and use of Artificial Intelligence (#52).

Participants emphasized the importance of designing OCCS that reflect youth preferences, behaviors, and digital habits. Services are increasingly integrating feedback loops and specific outreach via online community platforms and social media. Innovations such as reflective chatbots, flexible access formats, and youth councils were seen as effective ways to ensure relevance. One participant noted, “We launched a Discord server five years ago, and now it has 26,000 youth members. It’s their space—they decide what happens. Adults and professionals are present at set times to offer support, but then step back. This balance keeps youth engaged while ensuring help is available when needed.” (OCCS manager) This highlights the need to embed support within youth digital ecosystems.

Trust was described as foundational to OCCS effectiveness. Anonymity, transparency, and emotional validation were key trust-building mechanisms. Youth value being heard, respected, and given agency in their interactions. Services that clearly distinguish between human and AI responders, offer consistent follow-up, and empower youth to control their data and disclosures were seen as more credible.

The envisioned “gold standard” for OCCS includes hybrid human–AI collaboration, multilingual access, embedded services within youth platforms, and standardized quality assurance. Participants called for pan-European infrastructure, shared evaluation frameworks, and ethical, non-profit-led innovation. One participant described, “We run an annual self-assessment survey, but since it is not a formal accreditation, its validity is limited. That’s why we’re working on a more robust audit system to support improvement and ensure consistency across helplines.” (NGO project coordinator). This underscores the need for robust accountability mechanisms.

Adaptability was seen as essential for OCCS to remain effective amid technological, social, and policy shifts. Services are evolving through data-informed design, flexible staffing, and responsive training. Participants stressed the importance of adjusting language, metrics, and service formats to meet emerging needs. Crisis events, such as school shootings, were cited as examples of how OCCS must quickly pivot to address cascading emotional responses.

Support from AI tools (e.g., automated bots for translation and training counsellors) was viewed with cautious optimism. Participants explored its potential for triage, translation, training, and data analysis, while acknowledging risks around bias, emotional nuance, and legal accountability. Ethical deployment requires transparency, user choice, and human oversight—especially in crisis situations. This also applies to training automated bots that could support chat conversations. While some youth were perceived to prefer AI for its anonymity and responsiveness, others may feel unsafe or misunderstood. Services are experimenting with AI companions, training bots, and pre-chat intake tools but emphasized that AI should complement—not replace—human empathy.

## 4. Discussion

This study sought to provide consensus-based insights from diverse stakeholders to guide the design, evaluation, and implementation of OCCS for young people across Europe. Our findings underscore both the promise and complexity of OCCS. Participants highlighted their unique value in fostering emotional safety, accessibility, and trust, while also pointing to persistent challenges such as limited service availability, infrastructural fragility, and ethical tensions around anonymity and safeguarding. Importantly, the discussions emphasized that OCCSs are not a single, uniform solution: some operate as one-off, anonymous support options, while others are designed to integrate more closely with formal care pathways. Across countries and stakeholder groups, participants described remarkably similar challenges and opportunities, suggesting that OCCS share core structural features regardless of national context.

Building on this diversity, the following sections consider how stakeholder insights can inform the design of youth-friendly and inclusive OCCS models, the evaluation of services through adapted and context-sensitive frameworks, and the implementation of sustainable infrastructures that recognize different service philosophies and resource capacities.

### Reimagining Digital Support: The Promise and Complexity of Providing OCCS for Youth

Online counselling chat services (OCCS) occupy a unique and increasingly strategic position within the mental health care ecosystem. Situated on a continuum between informal support and formal primary care, OCCS can be conceptualized as part of the lower tiers of the WHO mental health service pyramid, which emphasizes accessible, community-based interventions as foundational to a comprehensive mental health system (World Health Organization, 2025). Their relatively low cost, high accessibility, and alignment with youth communication preferences position them as promising tools for early intervention, prevention, and triage.

However, existing evaluation frameworks—often designed for traditional or app-based digital interventions—do not fully capture their specific characteristics. As highlighted in our focus groups, OCCS challenge conventional metrics of effectiveness and usability. Their anonymous, text-based nature, flexible entry points, and reliance on relational dynamics call for a rethinking of how we assess digital mental health interventions. Traditional domains such as clinical validity, usability, and engagement remain relevant but must be adapted to reflect the unique capabilities and constraints of OCCS.

Despite their potential, few studies have rigorously evaluated the effectiveness of OCCS. A systematic review by Zhou et al. (2021) found that while online mental health interventions for youth are generally effective, only one of the 45 included randomized controlled trials (RCT) specifically examined synchronous chat-based counselling. The aforementioned RCT showed that web-based synchronous chat with mental health professionals led to significantly greater reductions in depressive symptoms in the intervention group compared to the waiting list condition (Kramer et al., 2014). Furthermore, Tibbs et al. (2022) noted that the literature on OCCS remains limited, with methodological challenges in evaluating real-time, anonymous interactions. However, recent research has begun to lay the groundwork for more targeted investigations. For example, Sindahl and van Dolen (2020) demonstrated that specific formal features of chat sessions—such as message density and session duration—are associated with improved outcomes, suggesting that the asynchronous nature of OCCS can still support meaningful therapeutic impact. Likewise, Sindahl et al. (2019, 2020) found that text-based counselling can positively affect well-being and empowerment, particularly when counsellors use child-centered strategies and empathetic communication. Bontinck et al. (2025) further showed that OCCS are well-suited to the needs of autistic individuals and their families, with high satisfaction and improved well-being reported immediately after chat sessions. These recent studies collectively underscore the emerging evidence base and point to promising directions for future research on OCCS effectiveness.

Theoretical models of psychotherapy suggest that three common factors—therapeutic alliance, expectancy, and health-promoting actions—are central to positive outcomes. Del Re et al. (2021) emphasize the role of therapist effects, showing consistent outcome differences across therapists regardless of modality. Applying these insights to OCCS raises several hypotheses: that text-based empathy and responsiveness can foster therapeutic bonds; that expectation-building may be achieved through clear, hopeful messaging; and that structured interventions (e.g., behavioral prompts or journaling) can encourage health-promoting actions. Future research should examine whether counsellor variability predicts outcomes in OCCS and whether early perceptions of empathy and alliance mediate improvements in mental health and well-being—even in the absence of visual or vocal cues. Analysis of chat conversations, potentially enhanced by AI-augmented text analysis tools (see e.g., Hitch, 2024), could also help address relevant research questions.

Accessibility and anonymity are among the most valued features of OCCS, particularly for youth who may be reluctant to seek traditional help, who prefer not to burden those close to them, or who feel uncomfortable asking sensitive questions to people they know (Takala et al., 2025). These features reinforce each other: anonymity lowers the threshold for engagement, while accessibility ensures that support is available when needed. Yet, significant gaps remain. Only 13% of OCCS in Belgium, Hungary, Spain, and Finland operate 24/7, despite evidence that a third of youth contacts occur after 8 p.m. Moreover, only 39% of services explicitly target vulnerable populations such as LGBTQ+ youth, neurodiverse youth, abuse victims, or migrants, and just 14% offer support in minority or foreign languages—despite this being a key recommendation of international quality frameworks (Higuera-Lozano et al., 2025). To address these gaps, OCCS providers could enhance cooperation among themselves, particularly between general and specially targeted services. While morning hours are burdensome for counsellors, it may be worthwhile to prioritize expanding service hours through hybrid models that combine human support with AI-driven chatbots. Additionally, investing in multilingual and culturally sensitive outreach can help ensure equitable access for all youth.

The ethical tension between safeguarding anonymity and ensuring safety is a recurring theme. Anonymity is essential for building trust, yet in crisis situations, breaching confidentiality may be necessary to prevent harm. Surani et al. (2023) highlight the importance of robust privacy protocols and secure infrastructure to manage this trade-off responsibly.

Additionally, the emotional toll on counsellors—especially when users disengage mid-chat or disappear without closure—should not be underestimated. Heavy reliance on volunteers, while cost-effective, raises concerns about continuity, supervision, and burnout. Ensuring sustainable staffing models and emotional support for counsellors is critical.

Infrastructure challenges also shape the viability of OCCS. While shared platforms among services (e.g., the Flemish model connecting over 20 chat services) offer opportunities for cost-sharing and warm referrals, they require sustained investment. Many OCCS providers are NGOs operating with limited budgets, often relying on free commercial tools that pose data security risks and limit interoperability. Only a minority use custom-built software designed for secure online counselling, and 23% still operate via social media (Higuera-Lozano et al., 2025). To strengthen infrastructure and ensure long-term sustainability, OCCS providers could collaborate within and between countries on developing secure, open-source chat platforms tailored to youth mental health needs, supported by pooled funding mechanisms and public–private partnerships.

Quality standards and systematic data collection are essential for service improvement, accountability, and funding advocacy. Yet, only 31% of OCCS providers report user numbers, and just 56% publish activity reports (Higuera-Lozano et al., 2025). This lack of data impedes efforts to evaluate impact, identify gaps, and attract resources. As Rees and Patalay (2019) argue, intentional data collection—whether through passive sensing, ecological momentary assessments, or administrative linkage—can transform mental health services by enabling personalized care and continuous improvement.

Finally, the role of artificial intelligence (AI) in OCCS is emerging. Currently, 9% of services in the studied countries integrate AI tools (Higuera-Lozano et al., 2025), but recent examples show the potential of chatbots to extend service hours and triage users effectively. AI has potential to support counsellors by suggesting responses, flagging crisis indicators, and analyzing usage patterns to optimize staffing. However, ethical deployment requires transparency, user consent, and human oversight—especially in high-risk situations—and should be restricted to secure, closed environments to protect user privacy.

Several limitations should be acknowledged. First, although the analysis captured substantial thematic convergence, additional stakeholder groups, such as policymakers, parents, and youth, might have identified different priorities not represented here. Second, the findings are based on qualitative data from focus groups conducted with participants from 6 European countries and the UK, as well as Gibraltar. While this approach allows for rich, context-sensitive insights, it may not capture the full diversity of OCCS models or youth experiences across Europe or globally. Future research employing larger, more diverse samples and mixed-method approaches could help address these limitations by capturing a broader range of OCCS models and youth experiences across different cultural and socio-economic contexts. Third, the study relied on purposive sampling through professional networks, which may have introduced selection bias toward more established or well-connected services. Fourth, while the coding process was rigorous and included reflexive validation and member checking, the interpretation of qualitative data remains inherently subjective. Finally, the study did not include direct input from youth users of OCCS, which limits the ability to assess user satisfaction, perceived impact, or unmet needs. Future research should incorporate youth perspectives through participatory methods and complement qualitative findings with quantitative outcome data to strengthen the evidence base for OCCS.

## 5. Conclusions

To conclude, this study highlights the evolving and diverse roles of OCCS in supporting youth mental health across Europe. Online counselling chat services vary widely in their aims, target groups, and resources: some function as one-off, anonymous support options, while others seek to integrate more closely into formal care pathways. Positioned on a continuum between informal and formal care, OCCS offer a low-threshold, youth-aligned, and scalable solution to bridge persistent gaps in access, especially for difficult-to-reach populations.

To translate our insights into action, several policy and practice implications emerge. From a policy perspective, European countries could strengthen OCCS by developing national or cross-national quality standards for digital youth counselling, promoting secure, interoperable, and open-source infrastructures, and ensuring sustainable funding models—for example, through pooled mechanisms or public–private partnerships. Policies should also encourage multilingual and inclusive service provision, given that only 14% of OCCS currently offer support in minority or foreign languages.

For clinical practice, counsellors and service providers should receive specialized training in text-based empathy, alliance building, and crisis management, recognizing the distinct relational dynamics of OCCS. Regular supervision and emotional support structures are needed to mitigate counsellor burden, particularly in volunteer-based models. Also, hybrid service models combining human support with ethically governed AI tools could extend availability while maintaining safety and trust. Across these implications, a central message emerges: OCCS should be understood not merely as technological solutions but as relational environments shaped by staff expertise, emotional labour, and organizational infrastructures. Recognizing this complexity is essential for designing policies that support both sustainable service delivery and meaningful youth engagement.

Future research should build on these findings through longitudinal and mixed-methods designs that combine chat-based text analyses with outcome and process data to identify mechanisms of change. Moreover, participatory research involving young service users is essential to capture lived experiences, evaluate perceived impact, and co-design next-generation OCCS that align with young people’s diverse needs and digital realities.

As digital mental health continues to expand, OCCS should be acknowledged not only as potential crisis tools but, for some models, as integral components of a broader, youth-centered mental health ecosystem. At the same time, it is important to recognize and respect the diversity of OCCS, ensuring that services with different aims and capacities are evaluated according to appropriate expectations. For those OCCS that explicitly aim to become part of structured service pathways, future research and policy should prioritize the development of tailored quality standards, cross-national collaboration, and the responsible integration of AI to enhance reach and responsiveness. By synthesizing practitioner perspectives across diverse European contexts, this study provides one of the first empirically grounded frameworks for strengthening and future-proofing OCCS, offering a strategic foundation for research, policy, and practice in digital youth mental health.

## Figures and Tables

**Table 1:** Characteristics of Participants in In-Person and Online Focus Groups.

Participant Number	Country	Professional Role	In-Person * (*n* = 27)	Online * (*n* = 23)
1.	Belgium	Chat-counselor	Yes	Yes
2.	Belgium	Youth mental health project officer	Yes	No
3.	Netherlands	NGO executive director	Yes	No
4.	Finland	OCCS manager	Yes	No
5.	Belgium	Trainer in online and blended support	Yes	No
6.	Spain	Chat-counselor	Yes	Yes
7.	Belgium	Master’s student	Yes	No
8.	Belgium	OCCS staff member	Yes	Yes
9.	Belgium	Student mentor	Yes	No
10.	Belgium	Chat-counselor	Yes	No
11.	Poland	Chat-counselor	Yes	No
12.	Belgium	OCCS staff member	Yes	No
13.	Netherlands	NGO project coordinator	Yes	Yes
14.	Belgium	Researcher	Yes	No
15.	Poland	Chat-counselor	Yes	No
16.	Finland	OCCS staff member	Yes	No
17.	Spain	Chat-counselor	Yes	No
18.	Hungary	Chat-counselor	Yes	No
19.	Spain	OCCS manager	No	Yes
20.	Belgium	Policymaker	No	Yes
21.	Finland	Researcher	No	Yes
22.	Gibraltar	OCCS manager	No	Yes
23.	Hungary	OCCS manager	No	Yes
24.	Hungary	Researcher	No	Yes
25.	Belgium	OCCS staff member	No	Yes
26.	Belgium	Researcher	No	Yes
27.	Belgium	Researcher	No	Yes
28.	Belgium	Researcher	No	Yes
29.	UK	OCCS manager	No	Yes
30.	Belgium	Researcher	Yes	Yes
31.	Belgium	Researcher	Yes	Yes
32.	Belgium	Researcher	Yes	Yes
33.	Finland	Researcher	Yes	Yes
34.	Finland	Researcher	Yes	Yes
35.	Spain	Researcher	Yes	Yes
36.	Spain	Researcher	Yes	No
37.	Hungary	Researcher	Yes	Yes
38.	Hungary	Researcher	Yes	Yes

Note. * Participation refers to whether the participant attended the in-person focus group or the online focus group.

**Table 2:** Categorization matrix for OCCS focus group analysis.

Main Category	Subtheme	Definition
Usability, accessibility & user engagement	Inclusive design	Features that support low-literacy, neurodiverse, or marginalized youth.
	Youth participation	Involving youth in co-design, feedback, or outreach.
	Stigma & misconceptions	Misunderstandings about who online chat services are for.
	User experience	Ease of use, satisfaction, and navigation.
	Communication preferences	How youth prefer to communicate and how that affects engagement in relation to online chat services.
Effectiveness & quality of service	Bonding & Alliance	How trust and emotional connection are built between youth and counsellors/professionals.
	Engagement & continuity	Maintaining user involvement over time, including repeat visits or follow-up.
	Training & Supervision	Preparation and ongoing support for counsellors/professionals to ensure quality in online chat services.
	Measuring impact	Assessing outcomes despite anonymity and one-off interactions.
	Ghosting & dropout	Users disengaging mid-conversation or not returning.
Infrastructure, integration & technical resilience	Technological innovation	New tools (not Artificial Intelligence) to enhance online chat services.
	Platform stability	Technical reliability, including connectivity and uptime.
	Interoperability	Ability to connect with other digital or clinical systems.
	Cross-national implementation	Challenges of deploying online chat services across Europe.
	Integration into care pathways	Linking online chat services with broader (mental) health services.
Sustainability & resource models	Funding models	Financial structures for long-term viability of online chat services.
	Volunteer engagement	Recruitment, retention, and support of volunteers or professionals working in online chat services.
	Staffing & capacity	Workforce availability and burnout or other issues in relation to professionals or counsellors in online chat services.
	Scalability	Expanding services to reach more users or regions.
	Pan-European collaboration	Shared infrastructure or standards across countries.
Ethical considerations, equity & crisis readiness	Anonymity vs. safety	Balancing privacy with emergency responses during chat conversations.
	Equity & inclusion	Fair access for all youth, including marginalized groups.
	Safeguarding protocols	Procedures for identifying and responding to risk and guaranteeing quality of service during online chat conversations.
	Data privacy & consent	Handling of personal data and informed consent.
Future vision	Youth-centered innovation	Keeping online chat services aligned with youth needs.
	Trust & credibility	Building and maintaining trust during chat conversations in online chat services.
	Long-term vision and ideal models	Aspirations for online chat services in 5–10 years. Description of a ‘gold standard’ for online chat services.
	Adaptability	Ability of online chat services to evolve with technological, social, or policy changes.
	Use of AI	Use of Artificial Intelligence (AI) in online chat services.

**Table 3:** Coding scheme and frequency of codes across focus groups and subthemes.

Main Category	Subtheme	FG 1	FG 2	FG 3	FG 4	FG 5	FG 6	FG 7	FG 8	In-Person Total (FG 1–4)	Online Total (FG 5–8)	Total
Usability, accessibility & user engagement	Inclusive design	6	11	8	1	6	4	4	3	26	17	43
	Youth participation	5	3	4	2	2	3	4	2	14	11	25
	Stigma & misconceptions	4	3	5	1	3	2	3	2	13	10	23
	User experience	6	5	10	3	6	5	5	3	24	19	43
	Communication preferences	5	5	6	3	3	3	4	4	19	14	33
Effectiveness & quality of service	Bonding & Alliance	5	6	5	8	4	7	2	4	24	17	41
	Engagement & continuity	8	3	4	4	9	2	3	5	19	19	38
	Training & Supervision	6	12	7	9	7	7	6	2	34	22	56
	Measuring impact	6	5	3	9	4	7	6	3	23	20	43
	Ghosting & dropout	3	2	3	1	4	3	0	4	9	11	20
Infrastructure, integration & technical resilience	Technological innovation	9	1	5	5	3	5	3	3	20	14	34
	Platform stability	1	3	4	1	2	0	1	2	9	5	14
	Interoperability	3	5	6	3	3	3	2	1	17	9	26
	Cross-national implementation	4	7	6	5	3	4	3	3	22	13	35
	Integration into care pathways	5	5	6	3	4	2	2	3	19	11	30
Sustainability & resource models	Funding models	4	5	4	4	2	4	2	1	17	9	26
	Volunteer engagement	4	11	9	6	4	8	4	1	30	17	47
	Staffing & capacity	4	4	7	4	2	7	2	2	19	13	32
	Scalability	4	5	6	2	4	4	1	3	17	12	29
	Pan-European collaboration	4	7	6	4	3	3	6	0	21	12	33
Ethical considerations, equity & crisis readiness	Anonymity vs. safety	7	6	6	3	4	1	2	4	22	11	33
	Equity & inclusion	5	6	12	1	3	4	3	2	24	12	36
	Safeguarding protocols	5	7	6	6	3	3	2	4	24	12	36
	Data privacy & consent	7	5	6	7	4	1	1	3	25	9	34
Future vision	Youth-centered innovation	2	2	10	3	3	2	3	4	17	12	29
	Trust & credibility	7	5	8	5	4	5	2	5	25	16	41
	Long-term vision and ideal models	6	2	8	5	4	0	3	1	21	8	29
	Adaptability	6	4	9	1	4	5	3	2	20	14	34
	Use of AI	10	8	7	11	2	8	3	3	36	16	52
Overall total		151	153	186	120	109	112	85	79	610	385	995

## Data Availability

Pseudonymized meta-data (e.g., coding schemes, logbook with actions related to data analysis) can be requested by e-mail (alexis.dewaele@ugent.be). Due to the private nature of the qualitative data, the latter are not available to third parties.

## Supplementary Materials

The following supporting information can be downloaded at https://www.mdpi.com/article/10.3390/ejihpe15120257/s1, Annex S1—Lay out focus groups & topic list; Annex S2—Categorization matrix; Annex S3—# Codes, Annex S4—Code Relations Browser.

## Abbreviations

The following abbreviations are used in this manuscript:

OCCS	Online Counselling Chat Services
AI	Artificial Intelligence
NGO	Non-Governmental Organization
CHAT-YOUTH	Crisis Help and Assistance for Youth during challenging Times
GDPR	General Data Protection Regulation
RCT	Randomized Controlled Trial
WHO	World Health Organization
