# Supplementary material for: Help Is Just a Message Away: Online Counselling Chat Services Bridging Gaps in Youth Mental Health?
Source: Eur J Investig Health Psychol Educ. 2025 Dec 15;15(12):257. doi: 10.3390/ejihpe15120257 (PMC12731582; doi:10.3390/ejihpe15120257)
Supplement: Supplementary file 1 [file ejihpe-15-00257-s001.zip › Annex S1 - Lay out focus groups & topic list.docx]

**Overall Objective**

To co-develop a **consensus statement** that is:

- **Practical**: grounded in real-world requirements and constraints
- **Useful**: applicable to policy, practice, and innovation
- **Integrated**: based on existing research, evaluation domains, and practice insights
- **Visionary**: forward-looking, with attention to future opportunities and challenges

**Discussion Structure (90 Minutes)**

**🔹 Part 1 – Setting the Scene (10 min)**

**Facilitator Introduction (2 min)**

- Briefly state the goals of the session
- Emphasize co-creation, integration of experience, evidence, and ambition

**Double check informed consent and set ground rules (6 min)**

**Framing Presentation (2 min)**

- Recap key elements from the presentation:
  - *Why chat counseling works*
  - *What needs to be evaluated*
  - *Requirements & challenges*
  - *Key future considerations*

**🔹 Part 2 – Breakout Topic Discussions (80 min total)**

Break into 6 groups (each with a moderator + note taker).
Each group tackles the first topic first, then the second and so on. Each group gets a different order of the topics to ensure that all topics are discussed but that time is allowed to have an in-depth discussion for a topic. At least two topics should be covered by each group.

**Discussion Topics & Moderator Guide**

**1. Clinical Effectiveness & Therapeutic Quality**

- How can we improve bonding, engagement, and continuity in OSCCS?
- What practices (training, supervision, integration) are critical for effectiveness?
- How can we assess and demonstrate impact, despite anonymity and ghosting?

**2. Usability, Accessibility & User Engagement**

- How can services become more inclusive (low-literacy, neurodiverse youth)?
- How can we involve young people more meaningfully in design and communication?
- How do we counteract misperceptions (“chat is only for serious problems”)?

**3. Infrastructure, Integration & Technical Resilience**

- What tech innovations are needed (e.g. AI, interoperability)?
- How do we secure stable infrastructure across different European contexts?
- How should OSCCS be embedded in broader care systems?

**4. Sustainability & Resource Models**

- What is needed to ensure stable, long-term funding?
- What role can volunteers play, and how do we keep them supported and engaged?
- What hybrid or pan-European models could increase sustainability?

**5. Ethical Considerations, Equity & Crisis Readiness**

- How can we respect anonymity *and* act during emergencies?
- What does ethical AI look like in the OSCCS context?
- How do we ensure equity across diverse groups and countries?

**6. Future Vision**

- What could/should OSCCS look like in 5–10 years?
- How do we keep services youth-relevant and trustworthy?
- What would a gold standard look like?

**🔹 Part 3 – Plenary Sharing & Drafting (5 min for each group)**

Each group shares 1–2 key **priorities or commitments** for the consensus statement.

Facilitator clusters input live (e.g., via whiteboard or slide) under the following headings:

- Minimum requirements
- Shared values
- Future ambitions
- Calls to action

**📝 Post-Session Output**

Facilitation team synthesizes input into a **1-page draft consensus statement** to be reviewed and refined collaboratively after the session.
