# Supplementary material for: Help Is Just a Message Away: Online Counselling Chat Services Bridging Gaps in Youth Mental Health?
Source: Eur J Investig Health Psychol Educ. 2025 Dec 15;15(12):257. doi: 10.3390/ejihpe15120257 (PMC12731582; doi:10.3390/ejihpe15120257)
Supplement: Supplementary file 1 [file ejihpe-15-00257-s001.zip › Annex S2 - Categorisation matrix.docx]

# Categorisation Matrix for OCCS Focus Group Analysis

| **Main Category** | **Subtheme** | **Definition** |
| --- | --- | --- |
| 1. Effectiveness & quality of service | Bonding & Alliance | How trust and emotional connection are built between youth and counsellors/professionals. |
| 1. Effectiveness & quality of service | Engagement & continuity | Maintaining user involvement over time, including repeat visits or follow-up. |
| 1. Effectiveness & quality of service | Training & Supervision | Preparation and ongoing support for counsellors/professionals to ensure quality in online chat services. |
| 1. Effectiveness & quality of service | Measuring impact | Assessing outcomes despite anonymity and one-off interactions. |
| 1. Effectiveness & quality of service | Ghosting & dropout | Users disengaging mid-conversation or not returning. |
| 1. Usability, accessibility & user engagement | Inclusive design | **Features** that support low-literacy, neurodiverse, or marginalized youth. |
| 1. Usability, accessibility & user engagement | Youth participation | Involving youth in co-design, feedback, or outreach. |
| 1. Usability, accessibility & user engagement | Stigma & misconceptions | Misunderstandings about who online chat services is for. |
| 1. Usability, accessibility & user engagement | User experience | Ease of use, satisfaction, and navigation. |
| 1. Usability, accessibility & user engagement | Communication preferences | How youth prefer to communicate and how that affects engagement in relation to online chat services. |
| 1. Infrastructure, integration & technical resilience | Technological innovation | New tools (not Artificial Intelligence) to enhance online chat services. |
| 1. Infrastructure, integration & technical resilience | Platform stability | Technical reliability, including connectivity and uptime. |
| 1. Infrastructure, integration & technical resilience | Interoperability | Ability to connect with other digital or clinical systems. |
| 1. Infrastructure, integration & technical resilience | Cross-national implementation | Challenges of deploying online chat services across Europe. |
| 1. Infrastructure, integration & technical resilience | Integration into care pathways | Linking online chat services with broader (mental) health services. |
| 1. Sustainability & resource models | Funding models | Financial structures for long-term viability of online chat services. |
| 1. Sustainability & resource models | Volunteer engagement | Recruitment, retention, and support of volunteers or professionals working in online chat services. |
| 1. Sustainability & resource models | Staffing & capacity | Workforce availability and burnout or other issues in relation to professionals or counsellors in online chat services. |
| 1. Sustainability & resource models | Scalability | Expanding services to reach more users or regions. |
| 1. Sustainability & resource models | Pan-European collaboration | Shared infrastructure or standards across countries. |
| 1. Ethical considerations, equity & crisis readiness | Anonymity vs. safety | Balancing privacy with emergency response during chat conversations. |
| 1. Ethical considerations, equity & crisis readiness | Equity & inclusion | Fair access for all youth, including marginalized groups. |
| 1. Ethical considerations, equity & crisis readiness | Safeguarding protocols | Procedures for identifying and responding to risk and guarantee quality of service during online chat conversations. |
| 1. Ethical considerations, equity & crisis readiness | Data privacy & consent | Handling of personal data and informed consent. |
| 1. Future vision | Youth-centered innovation | Keeping online chat services aligned with youth needs. |
| 1. Future vision | Trust & credibility | Building and maintaining youth trust during chat conversations in online chat services. |
| 1. Future vision | Long-term vision and ideal models | Aspirations for online chat services in 5–10 years. Description of a "gold standard" for online chat services. |
| 1. Future vision | Adaptability | Ability of online chat services to evolve with technological, social, or policy changes. |
| 1. Future vision | Use of AI | Use of Artificial Intelligence (AI) in online chat services. |
